# Diagnostic Performance of Serial High-Sensitivity Cardiac Troponin Measurements in the Emergency Setting

**DOI:** 10.3390/jcdd8080097

**Published:** 2021-08-13

**Authors:** Peter A. Kavsak, Mark K. Hewitt, Shawn E. Mondoux, Joshua O. Cerasuolo, Jinhui Ma, Natasha Clayton, Matthew McQueen, Lauren E. Griffith, Richard Perez, Hsien Seow, Craig Ainsworth, Dennis T. Ko, Andrew Worster

**Affiliations:** 1 Department of Pathology and Molecular Medicine, McMaster University, Hamilton, ON L8S 4L8, Canada; mcquemat@hhsc.ca; 2 Division of Emergency Medicine, McMaster University, Hamilton, ON L8S 4L8, Canada; mark.hewitt@medportal.ca (M.K.H.); shawn.e.mondoux@gmail.com (S.E.M.); worstea@mcmaster.ca (A.W.); 3 ICES McMaster, Faculty of Health Sciences, McMaster University, Hamilton, ON L8S 4K1, Canada; joshua.cerasuolo@ices.on.ca (J.O.C.); richard.perez@ices.on.ca (R.P.); seowh@mcmaster.ca (H.S.); 4 Department of Health Research Methods, Evidence, and Impact, McMaster University, Hamilton, ON L8S 4L8, Canada; maj26@mcmaster.ca (J.M.); griffith@mcmaster.ca (L.E.G.); 5 Department of Medicine, McMaster University, Hamilton, ON L8S 4L8, Canada; clayton@mcmaster.ca; 6 Division of Cardiology, McMaster University, Hamilton, ON L8S 4L8, Canada; ainswoc@mcmaster.ca; 7 ICES Central, Sunnybrook Health Sciences Centre, Toronto, ON M4N 3M5, Canada; dennis.ko@ices.on.ca

**Keywords:** death, myocardial infarction, high-sensitivity cardiac troponin, emergency department, diagnostic

## Abstract

Serial high-sensitivity cardiac troponin (hsTn) testing in the emergency department (ED) and the intensive cardiac care unit may assist physicians in ruling out or ruling in acute myocardial infarction (MI). There are three major algorithms proposed for high-sensitivity cardiac troponin I (hsTnI) using serial measurements while incorporating absolute concentration changes for MI or death following ED presentation. We sought to determine the diagnostic estimates of these three algorithms and if one was superior in two different Canadian ED patient cohorts with serial hsTnI measurements. An undifferentiated ED population (Cohort-1) and an ED population with symptoms suggestive of acute coronary syndrome (ACS; Cohort-2) were clinically managed with non-hsTn testing with the hsTnI testing performed in real-time with physicians blinded to these results (i.e., hsTnI not reported). The three algorithms evaluated were the European Society of Cardiology (ESC), the High-STEACS pathway, and the COMPASS-MI algorithm. The diagnostic estimates were derived for each algorithm for the 30-day MI/death outcome for the rule-out and rule-in arm in each cohort and compared to proposed diagnostic benchmarks (i.e., sensitivity ≥ 99.0% and specificity ≥ 90.0%) with 95% confidence intervals (CI). In Cohort-1 (*n* = 2966 patients, 15.3% had outcome) and Cohort-2 (*n* = 935 patients, 15.6% had outcome), the algorithm that obtained the highest sensitivity (97.8%; 95% CI: 96.0–98.9 and 98.6%; 95% CI: 95.1–99.8, respectively) in both cohorts was COMPASS-MI. Only Cohort-2 with both the ESC and COMPASS-MI algorithms exceeded the specificity benchmark (97.0%; 95% CI: 95.5–98.0 and 96.7%; 95% CI: 95.2–97.8, respectively). Patient selection for serial hsTnI testing will affect specificity estimates, with no algorithm achieving a sensitivity ≥ 99% for 30-day MI or death.

## 1. Introduction

Guidelines support the use of early and serial testing with high-sensitivity cardiac troponin (hsTn) in patients with symptoms suggestive of acute coronary syndrome (ACS) for the diagnosis of acute myocardial infarction (MI) [1,2,3]. Observational studies have assessed algorithms using low and high hsTn concentrations with respective minor and large differences in concentrations over a couple of hours in the emergency department (ED); these studies suggest hsTn alone may be sufficient to rule-out and rule-in MI [4,5]. However, not all studies have supported the safe rule-out or possible discharge of patients from the ED being assigned to the rule-out arms or low-risk categories with these shorter testing algorithms [6,7]. A recent randomized control trial assessing the European Society of Cardiology (ESC) 0/1 h algorithm further questions the safety of these algorithms as it did not reduce cardiovascular death or MI over the long term (2.7% composite outcome with standard protocol versus 3.3% with 0/1 h protocol; *p* < 0.001) [8].

The benchmark for safety estimates for ruling out MI is attributed to an international survey response from ED physicians that reported an acceptable 30-day miss-rate of major adverse cardiac events (MACE, defined as the following in the survey: “Most MACE’s are NSTEMI’s but there are also a small but significant number of others (death, cardiac arrest, cardiogenic shock, ventricular arrhythmia or AV block requiring intervention)” [9] would be ≤1% and “that clinicians may expect diagnostic strategies for the assessment of suspected ACS to achieve a sensitivity of 99% or higher for AMI or other MACE” [9]. Others have proposed predictive values be used [10], with the following diagnostic estimates being evaluated in studies: sensitivity ≥ 99% or negative predictive value (NPV) ≥ 99.5% for rule-out and specificity ≥ 90% or positive predictive value (PPV) ≥ 75% for rule-in [11,12,13]. Many algorithms have been developed and implemented based mainly on predictive values, and despite the number of published algorithms increasing, there are few head-to-head comparison studies performed in the same cohorts with even fewer algorithms validated in external populations to assess generalizability [3,10,14,15].

In the present study, we evaluated the three main published hsTnI algorithms that use serial measurements [3,5,14] in two different ED cohorts for 30-day MI or death (a common outcome reported by studies using these algorithms) [3,5,14]. In both cohorts, physicians were blinded to the hsTnI results with the cardiac troponin test being reported clinically belonging to a non-hsTn test version (note, standard, conventional or contemporary are terms that are also used to describe non-hsTn tests). The overall objective was to assess if the diagnostic estimates using these algorithms met any of the four diagnostic benchmarks (i.e., sensitivity ≥ 99%, NPV ≥ 99.5%, specificity ≥ 90%, PPV ≥ 75%). We also examined whether patients identified in the rule-out arms would also be classified as low-risk if the miss-rate for death or MI in these groups was ≤1% at 30 days.

## 2. Materials and Methods

The criteria from the standards for reporting diagnostic accuracy studies (STARD 2015) were followed [16], and the study was approved by the Hamilton Integrated Research Ethics Board (REB# 13-277 and 4717-D).

### 2.1. Study Design and Participants

**Figure 1:**
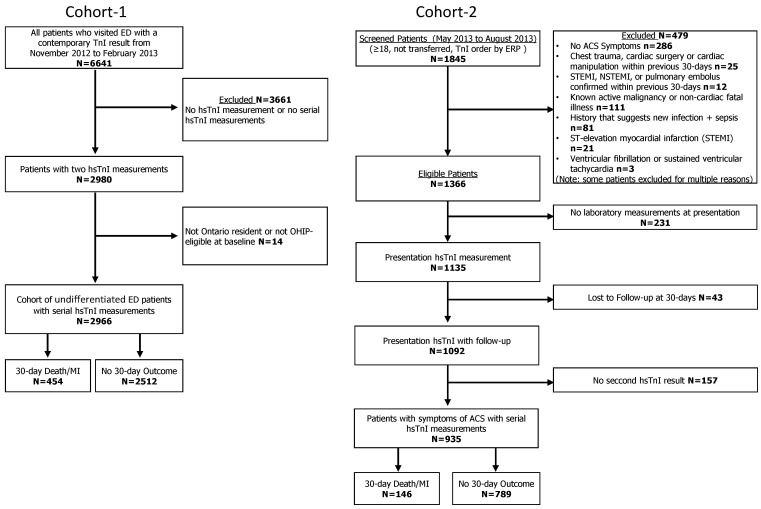
Flow diagram of the two cohorts.

In two different ED cohorts, we measured hsTnI for research purposes from the same blood samples used concurrently for testing with the non-hsTn assay that was used for clinical purposes. Both cohorts were observational studies and have been previously described using the presentation sample (i.e., the test results from the first sample measurement) [12,13,17,18]. The current retrospective analyses were confined to those participants with two hsTnI results to assess the algorithms (see Figure 1 flow of participants).

Cohort-1 consisted of consecutive patients who presented to the EDs of the Hamilton General Hospital, Juravinski Hospital, and St. Joseph’s Healthcare (Hamilton, ON, Canada) from November 2012 to February 2013 and had 2 hsTnI (blinded) results reported during their index ED visit. Patients who were not Ontario residents or covered by the Ontario Health Insurance Plan (OHIP) were excluded.

Cohort-2 was another observational cohort study (NCT01994577) conducted from May 2013 to August 2013 in patients presenting with symptoms suggestive of ACS to the ED at the same three Hamilton hospitals. Eligibility for enrollment included being an adult aged 18 or older, not being transferred from another hospital, with an ED physician having placed an order for cardiac troponin testing. Excluded participants were those patients with non-ACS symptoms (i.e., symptoms that did not include the following: chest pain/discomfort, pain or discomfort in one or both arms, pain or discomfort in the jaw, neck, back, pain or discomfort in the abdomen, shortness of breath, feeling dizzy or lightheaded, nausea and/or vomiting, diaphoresis, palpitations); or had ST-segment elevation MI at presentation; or had chest trauma, cardiac surgery or manipulation within 30-days of presentation; an MI (or pulmonary embolus) confirmed within the previous month; known active cancer or non-cardiac fatal illness; sepsis; ventricular fibrillation, or sustained ventricular tachycardia [17,19,20].

### 2.2. Health Outcomes

The primary outcome was 30-day MI or all-cause death [13,17]. In Cohort-1, past medical history and outcomes were obtained through linkages to administrative databases via ICES (formerly known as the Institute for Clinical Evaluative Sciences) using unique encrypted patient identifiers [13,18,21]. In Cohort-2, an emergency physician led an adjudication panel with the outcomes independently adjudicated by two members with disagreements not resolved by consensus referred to a third blinded reviewer [13,17]. From November 2012 to November 2014 in Hamilton, Ontario, the Abbott cardiac troponin I (non-hsTnI) assay was used, with the overall 99th percentile of 0.03 µg/L used to flag myocardial injury (i.e., evidence of biochemical myocardial injury present if cardiac troponin I concentrations ≥ 0.04 µg/L) [22].

### 2.3. Laboratory Testing and Algorithms Evaluated

The Abbott ARCHITECT hsTnI assay was measured in both cohorts with acceptable precision and performance documented for this assay [13,17]. In Cohort-1, there was no clinical recommendation on early sampling so a second sample was collected per standard care (reported median (25th to 75th) = 7.2 h (4.3 to 8.8) from presentation sample) [23]. In Cohort-2, there was a clinical recommendation to collect samples 3 h apart as per the Canadian Institutes of Health Research funded grant and study protocol [24]. As the diagnostic parameters have been published for the ESC 0/1 h algorithm cutoffs (sensitivity/NPV/specificity/PPV of 97.8%/99.4%/91.3%/50%) in Cohort-2 [24], the 0/2 h algorithm cutoffs were assessed (as the ESC recommends to use the 0/2 h algorithm as an alternative to the 0/1 h algorithm) [3], as well as the High-STEACS pathway [14] and COMPASS-MI algorithm [5] in both cohorts (see Table 1 for details of the algorithms).

For COMPASS-MI, physicians can select different cutoffs as detailed in the original publication and using the web-based calculator (https://compass-mi.com/ accessed on 26 April 2021) [5]. However, for a hospital system, a common rule-out and rule-in pathway is beneficial for achieving standardization of care. In consultation with emergency physicians and considering analytical performance, the following cutoffs and changes were used: rule-out if 1st sample hsTnI < 4 ng/L and change < 4 ng/L between the two samples and rule-in if 1st sample hsTnI ≥ 60 ng/L or change ≥ 18 ng/L between the two samples. The 4 ng/L cutoff can be measured with acceptable precision [25] and has been used in other studies for rule-out [13,17,26], with the change < 4 ng/L representing the total analytic error for hsTn at these low concentrations [27]. The 60 ng/L has been demonstrated to provide important prognostic information for 30-day cardiac events [28] and a change of 18 ng/L represents an analytically different result for assays with coefficient of variations ≤ 10% for concentrations < 60 ng/L. Applying these parameters, in the early sampling COMPASS-MI algorithm yielded the following diagnostic estimates based on the original publication [5]: for rule-out the sensitivity was 99.0% (95% CI: 98.3 to 99.5) and NPV was 99.7% (95% CI: 99.4 to 99.8), and for rule-in the specificity was 95.5% (95% CI: 95.0 to 96.0) and PPV was 75.8 (95% CI: 73.2 to 78.2). For both ESC and COMPASS-MI algorithms, there are three categories of risk: rule-out, rule-in and uncertain (observation group). For High-STEACS, there are only two categories of risk, low and high. The lower limit of detection (LoD) for the Abbott hsTnI assay ranges from 1.1 ng/L to 1.7 ng/L (see https://www.ifcc.org/media/478962/high-sensitivity-cardiac-troponin-i-and-t-assay-analytical-characteristics-designated-by-manufacturer-v042021.pdf, accessed on 5 August 2021). The lowest LoD of 1 ng/L was used in Cohort-2 with values < 1 ng/L converted to 0.9 ng/L; however, for Cohort-1 the numerical results reported from the instruments were used with the lowest concentration being 0 ng/L (no lower analytical limit or LoD was assigned to the instruments).

### 2.4. Statistical Analyses

We calculated non-parametric (i.e., median with 25th to 75th percentiles listed) ranges and frequencies (percentage, %) for the common variables of the two cohorts. We also calculated the diagnostic estimates sensitivity, NPV, specificity, PPV, and negative and positive likelihood ratios with 95% confidence intervals (CIs) of each cohort for both the rule-out and rule-in groups. We performed all statistical analyses using SAS 9.1.3 software (SAS Institute Inc, Cary, NC, USA), R software (R Foundation for Statistical Computing), and StatsDirect Statistical software. Acceptable performance of the algorithms was noted if the diagnostic estimates met or exceeded the stated benchmarks: sensitivity ≥ 99%, NPV ≥ 99.5%, specificity ≥ 90%, PPV ≥ 75%.

## 3. Results

Of the 2966 patients in Cohort-1, 454 patients (15.3%; 95% CI: 14.0 to 16.7) had the primary outcome (30-day MI or all-cause death), and of the 935 patients in Cohort-2, 146 patients (15.6%; 95% CI: 13.3 to 18.1) had the primary outcome with no significant difference in proportions (*p* = 0.82). There was no difference in the proportion of patients assigned to the uncertain (observation) group between the ESC and COMPASS-MI algorithms in Cohort-1 (*p* = 0.88) and Cohort-2 (*p* = 0.07). Additionally, in both cohorts, there was no difference in the frequency of women assigned to the various groups with the three algorithms; however, differences in the other variables associated with risk (i.e., clinical history) were evident (Table 2). There were, however, differences in time between samples collected between the two cohorts, with time between sample collection in Cohort-2 being shorter (median time = 3 h) (Table 3).

In Cohort-1, the High-STEACS pathway designated 1415 patients as rule-out (47%; 95% CI: 45.4 to 49.0%), with 4.0% (95% CI: 3.0 to 5.1) of this group having the primary outcome, whereas the COMPASS-MI algorithm designated the fewest patients (*n* = 697) as rule-out (23.6%; 95% CI: 22.0 to 25.1), with 1.4% (95% CI: 0.7 to 2.6) of this group having the primary outcome (Table 3). The proportion of patients in the rule-in group having the primary outcome was nearly identical between the ESC (39.2%; 95% CI: 25.9 to 42.6) and COMPASS-MI (39.6%; 95% CI: 36.2 to 43.0) algorithms and higher as compared to the High-STEACS pathway (25.7%; 95% CI: 23.5 to 27.9). Cohort-2 yielded similar estimates and trends (Table 3).

In Cohort-1, the highest sensitivity and NPV were observed with the COMPASS-MI rule-out arm (97.8%; 95% CI: 96.0 to 98.9 and 98.6%; 95% CI: 97.4 to 99.3, respectively), with the smallest negative likelihood ratio (0.08; 95% CI: 0.03 to 0.13) (Table 4). The highest specificity (80.3; 95% CI: 78.6 to 81.8), PPV (39.6%; 95% CI: 36.2 to 43.0), and positive likelihood ratio (3.63; 95% CI: 3.27 to 3.98) were also observed with the COMPASS-MI rule-in arm. In Cohort-2, the COMPASS-MI rule-out arm yielded the highest sensitivity (98.6%; 95% CI: 95.1 to 99.8), NPV (99.4%; 95% CI: 97.7 to 99.9), and lowest negative likelihood ratio (0.03; 95% CI: 0.01 to 0.13), with both the ESC and COMPASS-MI rule-in arms yielding estimates for specificity (>96%), PPV (>76%), and positive likelihood ratios (>17) (Table 4).

## 4. Interpretation

High-sensitivity cardiac troponin testing is the gold standard for detecting myocardial injury and aiding clinicians in making the diagnosis of MI [1,2,3]. An undetectable or low hsTn level within the normal reference interval is associated with a good prognosis and identifies patients at low risk for future cardiac events [29,30]. However, using a single low hsTn level to rule-out or discharge patients home may miss patients who are at high risk and have not reliably achieved a sensitivity ≥ 99.0%. Additional tools and laboratory tests could improve this performance (examples being HEART Score and Clinical Chemistry Score) [7,17]. Serial testing with hsTn may close this gap by identifying patients with evolving injury. This is the premise of the various algorithms that assess a low level with minimal change in concentration as being a good prognostic indicator and a high level with a large change as a poor prognostic indicator. However, these approaches are affected by analytical issues, thus requiring close collaboration between clinicians and the clinical laboratory to mitigate patient harm [31,32,33,34].

Our findings from assessing three well characterized algorithms demonstrate that for patients with symptoms suggestive of ACS, both the ESC and COMPASS-MI algorithms can identify high-risk patients as rule-in. However, we have also found that serial hsTnI alone cannot safely rule-out patients based on these proposed diagnostic benchmarks. The algorithm that yielded the highest sensitivity was COMPASS-MI, but the estimates (Cohort-1 sensitivity = 97.8% and Cohort-2 sensitivity = 98.6%) were slightly below 99.0% and below the derived estimates from the original publication [5]. These findings reaffirm the importance of assessing clinical signs and symptoms when using hsTn for early rule-out. On a population level, initial data on hsTn testing in Ontario (April 2013 to March 2017) were associated with lower MI, angina, and all-cause hospitalization during a time before the algorithms tested in this study were widely used [21]. These findings in Ontario are in agreement with the RAPID-TnT findings, where the standard protocol missed fewer outcomes as compared to the 0/1 h algorithm [8].

### 4.1. Limitations

First, our serial sampling occurred later than 2 h after presentation; however, recent data suggest a continuous linear release of hsTn levels in patients with MI [35]; thus, testing these algorithms with later samples (as performed in this analyses) would only improve the sensitivity estimates. Second, our findings are from a single urban city; however, the prevalence of outcomes is similar to other centers within North America [36]. Third, outcomes were assessed differently (i.e., adjudication versus databases); however, administrative databases, as used in Cohort-1, can be a robust source [37]. Fourth, different lower limits of reporting for hsTnI were utilized (0 ng/L in Cohort-1 and 0.9 ng/L in Cohort-2), leading to possible differences of 0.9 ng/L at the low end between the cohorts. However, acceptable variation of testing at the low end has been reported to be 0.8 ng/L, so differences of 1 ng/L could be based on different lower limits or the imprecision of testing alone [27].

### 4.2. Conclusions

**Figure 2:**
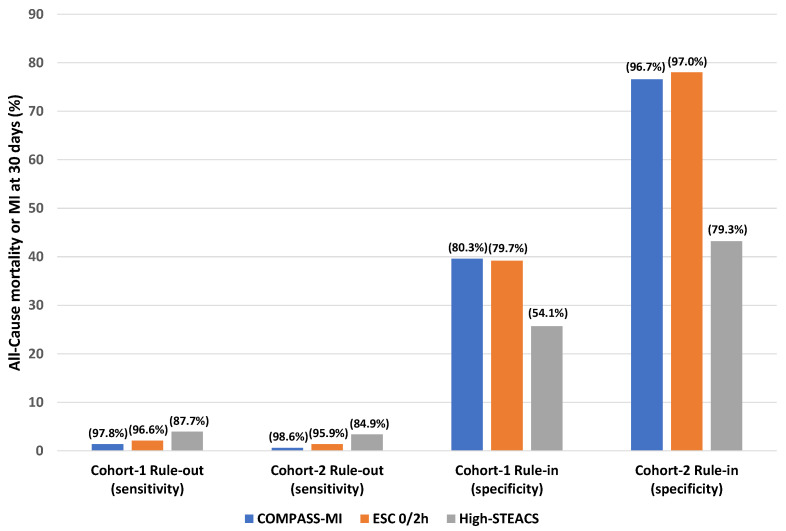
Summary figure of the performance of three different hsTnI algorithms for 30-day all-cause death or MI in undifferentiated ED patients with serial hsTnI measurements (Cohort-1) and ED patients with symptoms suggestive of ACS with serial hsTnI measurements (Cohort-2).

For patients with symptoms suggestive of ACS, both the ESC and COMPASS-MI algorithms can identify high-risk patients as rule-in (see Figure 2 for summary). However, no hsTnI algorithm alone can achieve the sensitivity or NPV estimates required for safely discharging patients as “rule-out MI”. Interventional studies assessing the addition of other laboratory or clinical variables in conjunction with hsTn are needed to determine the safest method of discharging patients home from the ED.

## Figures and Tables

**Table 1:** Algorithms used for rule-out and rule-in with hsTnI testing.

ESC 0/2 h algorithm	Rule-out: if 1st hsTnI < 6 ng/L and absolute change between 2nd and 1st sample < 2 ng/L	Rule-in: if 1st ≥ 64 ng/L or absolute change ≥ 15 ng/L between 2nd and 1st sample
COMPASS-MI algorithm	Rule-out: if 1st hsTnI < 4 ng/L and absolute change between 2nd and 1st sample < 4 ng/L	Rule-in: if 1st ≥ 60 ng/L or absolute change ≥ 18 ng/L between 2nd and 1st sample
High-STEACS	Rule-out: if 1st hsTnI ≤ sex-specific URLs and absolute change between 2nd and 1st sample < 3 ng/L	Rule-in: if 1st hsTnI > sex-specific URLs or absolute change between 2nd and 1st sample ≥ 3 ng/L

**Table 2:** Demographics and clinical history of the two ED cohorts characterized by the different rule-out and rule-in hsTnI algorithms.

Variable	Value	Cohort-1 (*n* = 2966)				Cohort-2 (*n* = 935)			
		Uncertain	Rule-In Group	Rule-Out Group	*p*-Value	Uncertain	Rule-In Group	Rule-Out Group	*p*-Value
ESC absolute criteria									
Number	Count	1264	837	865	-	401	109	425	-
Age (years)	Median (IQR)	77 (65–84)	78 (66–86)	60 (50–71)	<0.001	77 (64–85)	77 (63–85)	59 (48–70)	<0.001
Sex	Female (%)	612 (48.4%)	397 (47.4%)	453 (52.4%)	0.09	210 (52.4%)	49 (45.0%)	237 (55.8%)	0.12
History of arrhythmia	Count (%)	311 (24.6%)	203 (24.3%)	81 (9.4%)	<0.001	131 (32.7%)	39 (35.8%)	56 (13.2%)	<0.001
History of heart failure	Count (%)	417 (33.0%)	307 (36.7%)	93 (10.8%)	<0.001	114 (28.4%)	40(36.7%)	27 (6.4%)	<0.001
History of diabetes	Count (%)	509 (40.3%)	356 (42.5%)	220 (25.4%)	<0.001	137 (34.2%)	45 (41.3%)	92 (21.7%)	<0.001
History of myocardial infarction	Count (%)	217 (17.2%)	167 (20.0%)	61 (7.1%)	<0.001	175 (43.6%)	54 (49.5%)	109 (25.7%)	<0.001
COMPASS-MI absolute criteria									
Number	Count	1448	821	697	-	488	111	336	-
Age (years)	Median (IQR)	76 (65–84)	78 (66–86)	57 (48–67)	<0.001	76 (63–84)	77 (64–85)	56 (46–67)	<0.001
Sex	Female (%)	711 (49.1%)	386 (47.0%)	365 (52.4%)	0.113	266 (54.5%)	50 (45.1%)	180 (53.6%)	0.191
History of arrhythmia	Count (%)	340 (23.5%)	201 (24.5%)	54 (7.7%)	<0.001	142 (29.1%)	42 (37.8%)	42 (12.5%)	<0.001
History of heart failure	Count (%)	464 (32.0%)	300 (36.5%)	53 (7.6%)	<0.001	122 (25.0%)	43 (38.7%)	16 (4.8%)	<0.001
History of diabetes	Count (%)	566 (39.1%)	352 (42.9%)	167 (24.0%)	<0.001	156 (32.0%)	45 (40.5%)	73 (21.7%)	0.001
History of myocardial infarction	Count (%)	238 (16.4%)	157 (19.1%)	50 (7.2%)	<0.001	210 (43.0%)	53 (47.8%)	75 (22.3%)	<0.001
High-STEACS absolute criteria									
Number	Count		1551	1415	-		287	648	-
Age (years)	Median (IQR)		78 (66–85)	66 (54–78)	<0.001		78 (64–85)	64 (53–77)	<0.001
Sex	Female (%)		771 (49.7%)	691 (48.8%)	0.634		160 (55.8%)	336 (51.9%)	0.271
History of arrhythmia	Count (%)		374 (24.1%)	221 (15.6%)	<0.001		99 (34.5%)	127 (19.6%)	<0.001
History of heart failure	Count (%)		550 (35.5%)	267 (18.9%)	<0.001		97 (33.8%)	84 (13.0%)	<0.001
History of diabetes	Count (%)		646 (41.7%)	439 (31.0%)	<0.001		102 (35.5%)	172 (26.5%)	0.015
History of myocardial infarction	Count (%)		287 (18.5%)	158 (11.2%)	<0.001		136 (47.4%)	202 (31.2%)	<0.001

**Table 3:** The hs-cTnI levels and 30-day outcomes of the two ED cohorts characterized by the three hsTnI algorithms.

Variable	Value	Cohort-1 (*n* = 2966)				Cohort-2 (*n* = 935)			
		Uncertain	Rule-In Group	Rule-Out Group	*p*-Value	Uncertain	Rule-In Group	Rule-Out Group	*p*-Value
ESC absolute criteria									
Number	Count	1264	837	865	-	401	109	425	-
Time elapsed (hours) between 1st and 2nd hsTnI	Median (IQR)	8 (6–13)	8 (7–11)	6 (4–9)	<0.001	3 (3–3)	3 (3–3)	3 (3–3)	<0.001
1st hsTnI (ng/L)	Median (IQR)	13 (8–26)	89 (35–235)	3 (2–4)	<0.001	12 (7–23)	99 (41–229)	2 (1–4)	<0.001
2nd hsTnI (ng/L)	Median (IQR)	14 (9–26)	137 (67–492)	3 (2–4)	<0.001	13 (8–5)	188 (80–695)	2 (1–4)	<0.001
Absolute difference hsTnI (ng/L) between samples	Median (IQR)	3 (1–6)	56 (21–250)	1 (0–1)	<0.001	2 (1–3)	52 (15–288)	0 (0–1)	<0.001
All-cause mortality or MI at 30 days	Count (%)	108 (8.5%)	328 (39.2%)	18 (2.1%)	<0.001	55 (13.7%)	85 (78.0%)	6 (1.4%)	<0.001
COMPASS-MI absolute criteria									
Number	Count	1448	821	697	-	488	111	336	-
Time elapsed (hours) between 1st and 2nd hsTnI	Median (IQR)	8 (6–12)	8 (7–10)	6 (4–9)	<0.001	3 (3–3)	3 (3–3)	3 (3–3)	<0.001
1st hsTnI (ng/L)	Median (IQR)	11 (6–23)	93 (39–241)	2 (1–3)	<0.001	10 (5–19)	90 (50–229)	2 (1–3)	<0.001
2nd hsTnI (ng/L)	Median (IQR)	12 (7–23)	142 (72–506)	2 (2–3)	<0.001	11 (6–21)	182 (77–695)	2 (1–3)	<0.001
Absolute difference hsTnI (ng/L) between samples	Median (IQR)	2 (1–5)	56 (22–255)	1 (0–1)	<0.001	1 (0–3)	51 (11–288)	0 (0–1)	<0.001
All-cause mortality or MI at 30 days	Count (%)	119 (8.2%)	325 (39.6%)	10 (1.4%)	<0.001	59 (12.1%)	85 (76.6%)	2 (0.6%)	<0.001
High-STEACS absolute criteria									
Number	Count		1551	1415	-		287	648	-
Time elapsed (hours) between 1st and 2nd hsTnI	Median (IQR)		8 (7–12)	7 (4–10)	<0.001		3 (3–3)	3 (3–3)	<0.001
1st hsTnI (ng/L)	Median (IQR)		38 (16–100)	4 (2–8)	<0.001		30 (15–66)	3 (2–7)	<0.001
2nd hsTnI (ng/L)	Median (IQR)		48 (21–157)	5 (2–8)	<0.001		38 (21–100)	4 (2–7)	<0.001
Absolute difference hsTnI (ng/L) between samples	Median (IQR)		14 (5–64)	1 (0–1)	<0.001		6 (3–22)	1 (0–1)	<0.001
All-cause mortality or MI at 30 days	Count (%)		398 (25.7%)	56 (4.0%)	<0.001		124 (43.2%)	22 (3.4%)	<0.001

**Table 4:** Diagnostic performance of the different algorithms for death or MI at 30 days in the two ED cohorts.

Group	Sensitivity	Specificity	PPV	NPV	Positive LR	Negative LR	Sensitivity	Specificity	PPV	NPV	Positive LR	Negative LR
Cohort-1 ESC absolute criteria							Cohort-2 ESC absolute criteria					
Rule-out	96.0% (93.8–97.6%)	33.7% (31.9–35.6%)	20.8% (19.0–22.6%)	97.9% (96.7–98.8%)	1.45 (1.40–1.50)	0.12 (0.06–0.17)	95.9% (91.3–98.5%)	53.1% (49.6–56.6%)	27.5% (25.9–29.1%)	98.6% (97.0–99.4%)	2.04 (1.88–2.22)	0.08 (0.04–0.17)
Rule-in	72.2% (67.9–76.3%)	79.7% (78.1–81.3%)	39.2% (35.9–42.6%)	94.1% (93.0–95.0%)	3.57 (3.22–3.91)	0.35 (0.30–0.40)	58.2% (49.8–66.3%)	97.0% (95.5–98.0%)	78.0% (70.0–84.3%)	92.6% (91.2–93.3%)	19.14 (12.61–29.05)	0.43 (0.36–0.52)
Cohort-1 COMPASS-MI absolute criteria							Cohort-2 COMPASS-MI absolute criteria					
Rule-out	97.8% (96.0–98.9%)	27.3% (25.6–29.1%)	19.6% (18.0–21.3%)	98.6% (97.4–99.3%)	1.35 (1.31–1.38)	0.08 (0.03–0.13)	98.6% (95.1–99.8%)	42.3% (38.9–45.9%)	24.0% (22.9–25.2%)	99.4% (97.7–99.9%)	1.71 (1.61–1.82)	0.03 (0.01–0.13)
Rule-in	71.6% (67.2–75.7%)	80.3% (78.6–81.8%)	39.6% (36.2–43.0%)	94.0% (92.9–95.0%)	3.63 (3.27–3.98)	0.35 (0.30–0.41)	58.2% (49.8–66.3%)	96.7% (95.2–97.8%)	76.6% (68.6–83.0%)	92.6% (91.2–93.8%)	17.67 (11.82–26.41)	0.43 (0.36–0.52)
Cohort-1 High-STEACS absolute criteria							Cohort-2 High-STEACS absolute criteria					
Rule-out and Rule-in	87.7% (84.2–90.5%)	54.1% (52.1–56.1%)	25.7% (23.5–27.9%)	96.0% (94.9–97.0%)	1.91 (1.81–2.01)	0.23 (0.17–0.29)	84.9% (78.1–90.3%)	79.3% (76.4–82.1%)	43.2% (39.5–47.0%)	96.6% (95.1–97.7%)	4.11 (3.53–4.79)	0.19 (0.13–0.28)

## Data Availability

The studies were conducted before data sharing processes were in place, and thus individual data are not available. The data are not publicly available due to privacy.

## Trial Registration

ClinicalTrials.gov Identifiers: NCT01994577.

## Sample Availability

Not available.
